# Self-retained, cryopreserved amniotic membrane for a scleral defect caused by mitomycin C: A case report

**DOI:** 10.1016/j.ajoc.2024.102199

**Published:** 2024-10-18

**Authors:** Brett Bielory

**Affiliations:** a New York Eye and Ear Infirmary of Mount Sinai, New York, NY, USA; b Hackensack University Medical Center, Hackensack Meridian Health, Hackensack, NJ, USA; c Clara Maass Medical Center, Belleville, NJ, USA; d OPTUM Tri-State (NJ), formerly Riverside Medical Group, Rutherford, NJ, USA

**Keywords:** Amnion, Amniotic membrane, Cryopreserved, Melt, Mitomycin C, Sclera, Thinning

## Abstract

**Purpose:**

To report a case of scleral melting noted within weeks after symblepharon release and pterygium excision with peri-operative adjuvant topical Mitomycin C (MMC) that was salvaged with in-office cryopreserved membrane.

**Observations:**

A 61-year-old Hispanic gentleman with history of pterygium excision many years prior underwent right nasal pterygium excision and symblepharon release using bare sclera technique followed by topical MMC 0.1 % for a week, 16 years ago. He was noted to have a right nasal scleral thinning. He was successfully treated conservatively with in-office cryopreserved amniotic membrane without further progression of the scleral melting or surgical intervention required.

**Conclusions and Importance:**

Short-term complication of pterygium excision with adjuvant topical MMC may occur. This case shows that early detection and recognition of the complication can be sight-saving with in-office cryopreserved amniotic membrane.

## 1 Introduction

Scleral defects ranging from dellen, melts, thinning, scleromalacia and necrotizing scleritis are well known complications following pterygium surgery with adjunctive mitomycin C (MMC).^1^ These postoperative complications can lead to a number of further issues including perforation, uveal prolapse, endophthalmitis, and loss of the globe despite conventional treatments such as topical lubricating drops or ointment, and antibiotics and require surgical patch grafting.^2^, ^3^, ^4^

Cryopreserved amniotic membrane (AM) grafts have been used extensively in ophthalmology, including in the surgical setting for scleral melts with or without tenonplasty.^5^, ^6^, ^7^, ^8^ Herein, we describe the successful utilization of a self-retained AM in the in-office management of a severe scleral defect that developed after pterygium surgery.

## 2 Case report

**Fig. 1:**
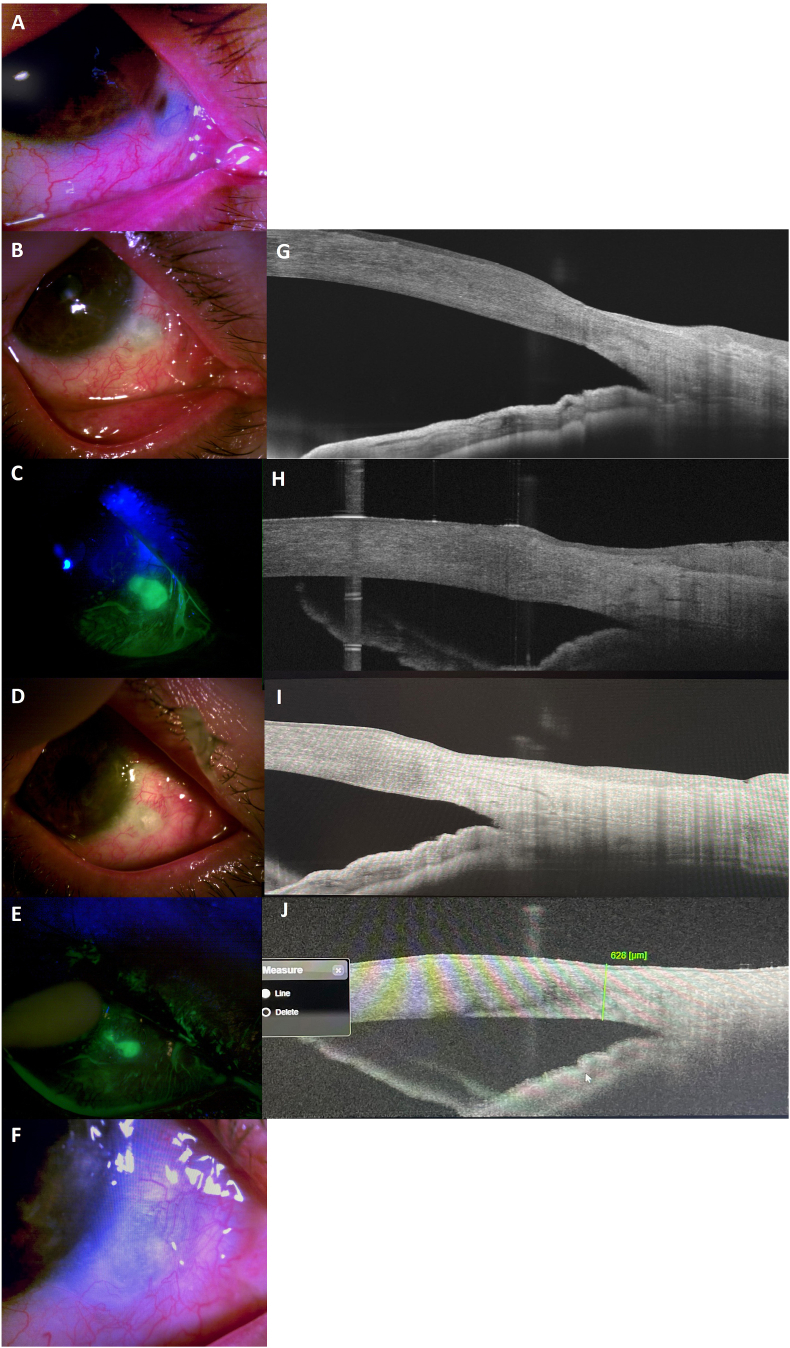
**External Photographic and AS-OCT Images of Scleral Defect before and after Treatment**. Patient presented with a scleral defect with uveal exposure at one-month post-pterygium excision surgery (A, G). There was notable healing after placement of self-retained cryopreserved AM as evidenced by lack of uveal exposure and reduced fluorescence staining at Day 5 (B, H), Day 12, (C, I), and Day 21 (E, J) days later. Cryopreserved AM allowed for revascularization of episcleral vessels to the area of scleral defect at day 30 (F).

A 57-year-old, Hispanic male with a prior history of previous pterygia surgery in his home country many years ago presented with complaints of bilateral (OD > OS) blurred vision, photophobia, ocular burning sensation, and chronic (>15 years) redness despite artificial tears and warm compress treatment. Examination revealed OD had Ocular Surface Disease Index (OSDI) score of 22, uncorrected visual acuity of 20/60, spherical equivalent 3.0 diopters, intraocular pressure (IOP) 14 mmHg, tear break-up time (TBUT) < 5 seconds, and central corneal thickness of 561um (pachymetry). Patient also noted visually significant diplopia on extreme temporal gaze (OD > OS). External examination revealed bilateral 3.5mm conjunctival pterygium, conjunctival injection (1+), corneal epithelial erosions (1–3+), inferonasal fornix shortening, and nasal symblepharon. A thorough discussion of the treatment options and their affiliated risks and benefits was performed. Due to the persistence and worsening of symptoms, surgery was elected with a decision to perform surgery on the right eye first due to worsening of clinical symptoms. The patient underwent uneventful symblepharon release and ocular surface reconstruction with excision of the pterygium and application of double-layered AM, double freeze cryotherapy, and 0.02 % of MMC for 45 seconds. No complications were noted on the 1- and 14-day follow-up visits. Patient returned one-month post-op with 20 % thinning and a bluish hue over the nasal sclera at the 4 o'clock limbus at the previous pterygium excision site (Fig. 1A). The patient denied any other symptoms except a headache for 2 weeks. Due to the proximity to the limbus and avoidance of further thinning, self-retained AM (Prokera Plus; BioTissue, Miami, Fl) was applied with concomitant use of 0.5 % moxifloxacin (Vigamox; Novartis, Basel, Switzerland) QID, neomycin polymyxin dexamethasone (Maxitrol; Novartis) qHS and 1 % prednisolone acetate (Pred Forte; Allergan, Dublin, Ireland) QID. After five days of wear, slit-lamp examination revealed conjunctival re-epithelization as evidenced by lack of uveal exposure (Fig. 1B and C). Anterior segment optical coherence tomography (AS-OCT; TOPCON, Oakland, NJ) also demonstrated the thickness of the sclera increased from 417μm to 739μm (Fig. 1G and H). Additional application of AM was applied on day 7, 14, and 21. The clinical appearance continued to progress at 12 and 21 days after initial scleral defect diagnosis, and the sclera appeared regularly thick and white in color covered by conjunctiva with a staining and non-pooling epithelial defect that was closed and re-epithelialized at day 30 (Fig. 1D–F). The thinnest sclera thickness was measured to be 727μm and 628μm at 12 and 21 days, respectively (Fig. 1I and J). The visual acuity improved to 20/30 while all symptoms resolved. The condition remained stable without signs of inflammation for an additional 3 months without any further surgical intervention.

## 3 Discussion

This case report that topical placement of self-retained cryopreserved AM in-office can be an efficient way to address scleral defects after pterygium surgery. Expeditious treatment led to visual recovery, improved scleral thickness, and complete epithelialization. Outcomes, including re-vascularization, are consistent with those reported with use of AM in the surgical setting for scleral melts with tenonplasty,^5^ albeit utilization of self-retained AM facilities faster care and avoidance of surgical intervention. The clinical benefits of AM for scleral defects may be due to its anti-inflammatory properties, as its been shown to reduce infiltration of pro-inflammatory cells into the wound site, induce cell death of activated neutrophils, and polarize macrophages toward anti-inflammatory phenotype which may modulate ongoing inflammation and promote wound healing.^9^

## 4 Conclusion

Collectively, this case highlights the in-office management of a scleral defect with self-retained AM to facilitate recovery of the ocular surface.

## Patient consent

Written consent to publish deidentified medical information and clinical photographs was obtained from the patient. This report does not contain any personal information that could lead to the identification of the patient.

## Conflicts of interest

Speaker for BioTissue.

## Authorship

All authors attest that they meet the current ICMJE criteria for Authorship.

## Funding

None.

## Declaration of competing interest

The authors declare that they have no known competing financial interests or personal relationships that could have appeared to influence the work reported in this paper.
